# Parent-child math anxiety and math-gender stereotypes predict adolescents' math education outcomes

**DOI:** 10.3389/fpsyg.2015.01597

**Published:** 2015-11-03

**Authors:** Bettina J. Casad, Patricia Hale, Faye L. Wachs

**Affiliations:** ^1^Department of Psychological Sciences, University of Missouri-St. LouisSt. Louis, MO, USA; ^2^Department of Mathematics and Statistics, California State Polytechnic University, PomonaPomona, CA, USA

**Keywords:** math anxiety, gender stereotypes, gender, parents, math education

## Abstract

Two studies examined social determinants of adolescents' math anxiety including parents' own math anxiety and children's endorsement of math-gender stereotypes. In Study 1, parent-child dyads were surveyed and the interaction between parent and child math anxiety was examined, with an eye to same- and other-gender dyads. Results indicate that parent's math anxiety interacts with daughters' and sons' anxiety to predict math self-efficacy, GPA, behavioral intentions, math attitudes, and math devaluing. Parents with lower math anxiety showed a positive relationship to children's math outcomes when children also had lower anxiety. The strongest relationships were found with same-gender dyads, particularly Mother-Daughter dyads. Study 2 showed that endorsement of math-gender stereotypes predicts math anxiety (and not vice versa) for performance beliefs and outcomes (self-efficacy and GPA). Further, math anxiety fully mediated the relationship between gender stereotypes and math self-efficacy for girls and boys, and for boys with GPA. These findings address gaps in the literature on the role of parents' math anxiety in the effects of children's math anxiety and math anxiety as a mechanism affecting performance. Results have implications for interventions on parents' math anxiety and dispelling gender stereotypes in math classrooms.

## Introduction

The status of math education in the US is cause for concern. Standardized math test performance indicates the US is ranked 35th out of 64 countries (National Center for Education Statistics, 2012). Politicians, educators, and researchers may point the blame at the US system of higher education, but another source should share the blame: our math-phobic culture (Burns, 1998; Chew and Dillon, 2014). Many Americans report fear or anxiety about conducting math and many students shy away from math-intensive disciplines such as the sciences, technology (e.g., computer science), engineering, and of course, mathematics and statistics (STEM; Meece et al., 1990; Chipman et al., 1992). Many Americans report that they just do not like math and statistics. This is problematic as mathematics is a gateway field for STEM disciplines and societal advancement in technology and science (Roman, 2004). If the majority of Americans are afraid of math, as a country we face falling further behind our math-friendly counterparts.

This social problem leads researchers and educators to ask why, where does this math anxiety originate? Math anxiety can be defined as “feelings of fear, apprehension, or dread that many people experience when they are in situations that require solving math problems” (Maloney et al., 2014, p. 404). Research on adolescents' math anxiety has pointed to parents, teachers, and peers as major environmental sources (Wigfield and Eccles, 2000; Beilock et al., 2010; Gunderson et al., 2012). We learn our math phobia in part from important others who influence our early life development. Another potential source of math phobia that has received research attention is cultural stereotypes about gender and math (Steffens et al., 2010; Cheryan, 2012). Despite evidence that the gender gap in math performance in the US has disappeared (Hyde et al., 2008; Lindberg et al., 2010), cultural biases about the superiority of boys and men in math permeate our social consciousness. Media attention has proscribed disparaging comments about girls' and women's inferiority in math and science (e.g., former Harvard President Lawrence Summers and Nobel Laureate Tim Hunt; Bombardieri, 2005; Associated Press, 2015). Yet, these cultural influences have lasting effects and will likely remain pervasive for years to come, as research shows stereotype change is a slow process (Devine and Elliot, 1995) and public attitudes are slow to change following cultural shifts (e.g., Civil Rights Movement and school desegregation; Newport et al., 1999).

The purpose of the present studies is to further probe the social determinants of adolescents' math anxiety by examining the relationship between math anxiety from same and other-gender parents with children's math anxiety and how this relates to math education outcomes. Parents' math anxiety is conceptualized as a moderator, determining the strength and direction of the relationships between children's math anxiety and math education outcomes. A plethora of research has examined the relationship between parents' (primarily mothers') math-gender stereotypes and perceptions of their child's math abilities with children's math attitudes and math anxiety (Eccles and Jacobs, 1986; Yee and Eccles, 1988; Midgley et al., 1989; Eccles et al., 1990; Jacobs, 1991, 2005; Jacobs and Eccles, 1992); however, little research has examined parents' own math anxieties (Gunderson et al., 2012; see Maloney et al., 2015). Addressing this gap in the literature, this study examines parents' own math anxiety and how it interacts with children's math anxiety to predict math education outcomes. Further, the first study examines these relationships within same- and mixed-gender parent-child dyads to explore the gendered nature of the intergenerational transmission of math anxiety (e.g., see for example Gniewosz and Noack, 2012). Addressing a call for a mechanistic approach (Gunderson et al., 2012), the second study examines children's math-related gender stereotypes as a source of math anxiety and tests math anxiety as a mechanism through which math-gender stereotypes negatively influence math outcomes for *both* girls and boys. The math education outcomes examined herein include math self-efficacy, math attitudes, math devaluing, math education behavioral intentions, and classroom math performance (GPA).

### Teachers and parents as sources of math anxiety

Eccles et al. (1983) developed an Expectancy-Value Theory of achievement motivation, originally in mathematics, that describes various cultural, social, interpersonal, and individual factors that influence children's motivations, task values, expectations for success, and achievement related choices. Included in the cultural milieu factors are cultural stereotypes about the subject and occupation (e.g., mathematics) and socializers' (e.g., parents) beliefs and behaviors, as well as children's own perceptions of socializers' beliefs and society's stereotypes about the domain. The Expectancy-Value Theory has received extensive support over the past few decades (see Wigfield and Eccles, 2000, for a review). Thus, the role of parents in influencing children's values, beliefs, expectations, performance, and choice in the math domain is well-known.

Both teachers and parents play a major role in socializing children's academic values and attitudes and an extensive body of research documents how parents' and teachers' expectations, gender stereotypes, and attributions impact children's math attitudes and performance (Yee and Eccles, 1988; Eccles et al., 1990; Tiedemann, 2000; Jacobs, 2005). Interestingly, however, little research has examined teachers' and parents' own math anxiety as an antecedent for children's math anxiety, attitudes, and achievement. A recent study found that female teachers' math anxiety impacted early elementary school girls', but not boys', math achievement and attitudes (Beilock et al., 2010). Specifically, girls whose teacher had higher math anxiety had lower math grades and learned less content at the end of the year compared to girls whose teacher had lower math anxiety, even after controlling for girls' math achievement in the beginning of the school year. It seems math-anxious teachers reinforced math-gender stereotypes as girls' endorsement of math-gender stereotypes mediated the effect of teacher anxiety on their math performance. A study with second grade elementary children examined parents' math anxiety in relationship to children's math anxiety, activities, and academic self-perceptions, but found no effects (Jameson, 2014). This work suggests the need for continued investigation of the role of parents' anxiety in children's anxiety in various developmental stages to determine when this effect begins. The only known study to find an effect of parents' math anxiety on their first and second grade child's math anxiety is a recently published study (Maloney et al., 2015). Thus, the present study focuses on parents' math anxiety as a socializing agent of children's math anxiety and the downstream effects on math education outcomes.

One reason researchers may have ignored the role of parents' anxiety in developing children's math anxiety and performance is a common (mis)perception that math learning is more likely to take place during school than at home and the role of parents may be less critical than teachers in math learning (Cannon and Ginsburg, 2008). However, just as teachers serve as role models for students, parents serve as long-term role models and their beliefs can influence their children as children develop their own identities, values, and efficacy (Yee and Eccles, 1988; Eccles et al., 1990; Tiedemann, 2000; Jacobs, 2005). Another potential reason for this gap in the literature is that people may not think about parents computing math, unless it is part of their occupation (e.g., accounting, banking). In contrast, school is the domain in which math is learned and regularly used and teachers perform mathematical problems publically in front of students and therefore seem to have more direct influence on children's math attitudes and anxiety. However, parents, regardless of their profession, likely perform more mundane mathematical computations on a regular basis such as making a household budget, balancing a checkbook, and calculating a tip at a restaurant, which can elicit math anxiety (Ashcraft, 2002). Parents' math anxiety may be subtly communicated to children through these mundane activities, or even more directly in their role of helping (or not helping) children with their math homework (Bhanot and Jovanovic, 2005; Maloney et al., 2015).

### Does parent gender matter?

Beilock et al. (2010) found that female teachers' math anxiety affected girls' math anxiety, performance, and amount of math learning over a school year, but it did not affect boys. This raises the question of the gender dynamics of math attitude, anxiety, and math-gender stereotype transmission between teachers and students and parents and children. Beilock et al. (2010) did not examine effects of male teachers, likely because more than 90% of elementary school teachers are female, and this is a new area of research, thus the effect of teacher gender remains a question for investigation. Likewise, only one published study has examined the effect of parents' math anxiety on children's math anxiety and achievement (Maloney et al., 2015), but sample demographics did not allow for a gender analysis, thus the question of parent gender is also yet to be addressed.

A great deal of the research using an expectancy-value framework focuses on the transmission of math-gender stereotypes from parents to children and how stereotypes influence children's achievement outcomes. This body of work has examined the gender of parents, particularly in the transmission of their math-gender stereotypes and math attitudes, but not specifically their math anxiety. This literature can inform questions and hypotheses about the gender composition of parent-child dyads in the intergenerational transmission of math anxiety. For example, is the influence of mothers to daughters and fathers to sons greater than mixed-gender transmission of mothers to sons and fathers to daughters? Research has mostly supported the same-gender dyad model such that mothers in particular communicate math-gender stereotypes to their daughters (O'Bryan et al., 2004), which subsequently predicts daughters' academic and career choices, even several years later (Bleeker and Jacobs, 2004). For example, if mothers endorse math-gender stereotypes that men are superior to women in math, they may communicate this (intentionally or unintentionally) to their daughters, who then may show less interest in math and choose other academic and career domains. Indeed, girls' and women's choices of academic and career trajectories is one explanation for the underrepresentation of women in STEM, rather than a lack of ability explanation (Wang et al., 2013). Many girls and women who show high aptitude in multiple domains choose academic and career paths outside of STEM in part because they have more opportunities available to them (Wang et al., 2013).

There are several reasons we hypothesize that same-gender parent-child transmission of math anxiety is more common than mixed-gender transmission. First, women and girls tend to experience greater math anxiety than men and boys (Hembree, 1990; Ramirez et al., 2013), regardless of their actual math ability (Hyde et al., 1990; Meece et al., 1990; Devine et al., 2012), and it begins as early as first and second grade (Harari et al., 2013; Ramirez et al., 2013) and increases as children get older (Hembree, 1990). Currently about 20% of the population is characterized as high in math anxiety (Eden et al., 2013). If women are more likely to suffer from math anxiety than men, then it is logical to predict that mothers experience greater math anxiety than fathers and likely communicate this to their children, particularly daughters, who also are more likely to have high math anxiety than sons. Second, gender role socialization most commonly occurs with same-gender caregivers (Bussey and Bandura, 1984). During development, daughters may be more likely to pick up on mothers' math anxiety than fathers', and sons from fathers rather than mothers. Of course there are children who strongly identify with other-gender parents and may more quickly adopt their beliefs, values, and attitudes (e.g., daddy's girl or mama's boy; Gniewosz and Noack, 2012). Finally, parents often hold gender stereotypes about their children's performance in math, believing that sons' have stronger math ability than daughters, even when there is no evidence to support this belief (Furnham et al., 2002). As a result, parents may expect daughters to perform more poorly in math, which may contribute to greater math anxiety for girls.

### Antecedents and effects of math anxiety

There is a large body of literature documenting the negative effects of math anxiety; however, there is still much we do not know. The majority of research focuses on negative consequences rather than antecedents and contexts in which math anxiety develops (Ashcraft and Ridley, 2005; Jameson, 2014; Maloney et al., 2015), and more research is needed with children (Jameson, 2014). Recent research has documented cognitive and biological antecedents of math anxiety including diminished working memory capacity, low math ability, attentional bias, and genetic factors (Wang et al., 2014; Suárez-Pellicioni et al., 2015).

The present studies examine an environmental factor, specifically parents' anxiety as a moderator of adolescents' math anxiety, thus helping to fill this gap in the literature.

Several negative consequences of math anxiety include avoidance of mathematics (e.g., math courses and math-intensive careers), less confidence, lower math self-efficacy, and more negative attitudes toward math (Hembree, 1990; Ashcraft et al., 1998; Ashcraft, 2002). Avoidance of math courses and math-intensive careers may be one explanation for the gender gap in STEM careers (Wigfield and Eccles, 2000; Gunderson et al., 2011; Cheryan, 2012). Math anxiety causes lower math performance, regardless of actual math ability (Hembree, 1990; Maloney and Beilock, 2012; Park et al., 2014).

Another negative consequence of math anxiety is low math self-efficacy. Self-efficacy is the confidence that one has the ability to succeed in the domain (Schunk, 1981, 1982a,b; Pajares, 1996). Although research clearly demonstrates the importance of math self-efficacy in math achievement and attitudes, few studies have examined how self-efficacy relates to math anxiety (Jameson, 2014). Two studies have found a negative relationship between math self-efficacy and math anxiety (Meece et al., 1990; Cooper and Robinson, 1991). Math anxiety was directly related to both boys' and girls' math ability perceptions, but interestingly, not to math grades (Meece et al., 1990). Given the importance of reducing students' math anxiety in order to promote positive math achievement and attitudes, many studies have examined ways that parents can help reduce children's math anxiety (e.g., Vukovic et al., 2013).

### Overview of studies

The current studies address different gaps in the literature by exploring antecedents of math anxiety: parents' math anxiety (Study 1) and math-gender stereotypes (Study 2), and a mechanistic (mediational) perspective of math anxiety (Study 2). Study 1 posits an interaction between parent and children's math anxiety in predicting several math education outcomes including math self-efficacy, math attitudes, math devaluing, math education behavioral intentions, and math GPA. These relationships are tested within the dyadic relationships of parents and children and the gender of parent and child dyad is examined for similar and different patterns in math education outcomes. Study 2 examines children's endorsement of math-gender stereotypes as an antecedent of math anxiety, and tests math anxiety as a mechanism through which math-gender stereotypes negatively influence math outcomes for *both* girls and boys.

## Study 1

### Hypotheses

Based on the literature reviewed we predict (1) main effects of child's math anxiety on the outcome variables. Specifically, greater math anxiety will predict (1a) lower math self-efficacy, (1b) lower math GPA, (1c) lower math education behavioral intentions, (1d) more negative math attitudes, and (1e) greater math devaluing. We predict (2) an interaction between parents' and children's math anxiety such that higher levels of children's math anxiety will be negatively correlated with math education outcomes, and the correlations will be strongest when parents also have higher math anxiety. Finally, we predict that (3) same-gender parent-math dyads are most likely to show significant relationships between math anxiety and education outcomes (both positive and negative), particularly (3a) mother-daughter dyads. Relatedly, (3b) we expect mothers and daughters to show patterns indicative of higher anxiety than other parent-child dyads. We expect (3c) few if any significant relationships for mixed-gender dyads including mother-son and father-daughter.

### Method

#### Participants

A total of 1342 parents were recruited and 683 participated^1^, resulting in a 51% response rate. Student participants included 377 (55%) girls and 306 (45%) boys in 6th (*n* = 157, 23%), 7th (*n* = 291, 43%), or 8th (*n* = 235, 34%) grade honors (*n* = 366, 55%) or standard math (*n* = 298, 45%) classes (e.g., Algebra Readiness, Pre-Algebra, or Algebra)^2^. Students' ages ranged from 11 to 14 reflecting ages in the 6th through 8th grades. There were 8 middle schools from southern California with 24 math teachers participating. Dyad types consisted of Mother-Daughter (*n* = 315, 46%), Mother-Son (*n* = 239, 35%), Father-Daughter (*n* = 62, 9%), and Father-Son (*n* = 67, 10%). The majority of students (*n* = 538, 88%) were born in the US. In contrast, the majority of parents were born outside the US (62%, *n* = 419). The majority of students and parents born outside the US were born in Mexico, South America, or an Asian country (e.g., China, Cambodia, Vietnam, Philippines). Students' self-reported race/ethnicity included 66% (*n* = 435) Latino/a or Hispanic, 10% (*n* = 62) Asian/Pacific Islander, 9% (*n* = 58) multiracial, and less than 5% each of Black/African American, Native American, White/Caucasian, or other. Parents' self-reported race/ethnicity included Latino/a or Hispanic 73% (*n* = 486), 10% (*n* = 65) Asian/Pacific Islander, 7% (*n* = 47) White/Caucasian, 7% (*n* = 45) Black/African American, and less than 5% of each group Native American, multiracial, or other. Parents' ages ranged from 22 to 63 (*M* = 42.20, *SD* = 6.73). Parents' education and household income are shown in Table 1.

**Table 1:** **Parents' education and household income**.

**Level of education**	**Mother *N* = 525**	**Father *N* = 121**	**Household income (in thousands)**	**Mother *N* = 211**	**Father *N* = 72**
8th Grade or less	13.9%	8.3%	<5	20.4%	8.3%
9th–12th Grade	15.8%	14.0%	5–9999	5.2%	1.4%
HS Graduate	23.2%	20.7%	10–14,999	13.7%	11.1%
Some College	28.0%	26.4%	15–24,999	15.6%	5.6%
College Graduate	14.7%	22.3%	25–34,999	21.8%	13.9%
Post Graduate	4.4%	8.3%	35–49,999	9.5%	22.2%
Missing information (excluded from calculations)	5.2%	6.2%	50–74,999	8.1%	22.2%
			75 or more	5.6%	15.3%
			Missing Information (excluded from calculations)	61.9%	44.2%

*Values are rounded to one decimal place for ease of reading. HS, High school education; Some College, attended college but did not complete a degree. Income is household income in thousands per year*.

#### Materials

The students' questionnaires contained items assessing math anxiety, math self-efficacy, math education behavioral intentions, math attitudes, and math devaluing. Math class GPA was obtained directly from the teacher. Parents' questionnaires contained a variety of similar measures, but only parents' math anxiety data are reported here.

Children's math anxiety was assessed by 3 items rated on a scale from 1 (*Very Strongly Disagree*) to 6 (*Very Strongly Agree;* based on Marx and Stapel, 2006). Items included “I often get nervous when I have to do math,” “Many times when I see a math problem I just ‘freeze up’,” and “I have never been as good in math as I am in other classes.” The items had acceptable internal consistency (α = 0.731) and were averaged so that higher values represented greater anxiety.

Parents' math anxiety was assessed by 2 items rated on a scale from 1 (*Very Strongly Disagree*) to 6 (*Very Strongly Agree;* based on Marx and Stapel, 2006). Items included “Many times when I see a math problem I just ‘freeze up’,” and “I have never been as good in math as in other classes in high school.” The items were moderately to highly correlated, *r*_(558)_ = 0.548, *p* = 0.001 and were averaged so that higher values represented greater anxiety.

Children's math self-efficacy was measured by 5 items rated on a scale from 1 (*Not at all Confident*) to 6 (*Very Confident;* based on Zimmerman and Martinez-Pons, 1988). Sample items included “How confident are you that you will pass your math class at the end of the term?” “How confident are you that you will pass math at the end of this term with a grade better than a B?” and “How confident are you that you will get an A?” The items had acceptable internal consistency (α = 0.898) and were averaged so that higher values represented greater math self-efficacy.

Children's math education behavioral intentions were measured by 6 items rated on a scale from 1 (*Very Strongly Disagree*) to 6 (*Very Strongly Agree;* adapted from Sparks et al., 1997; Butler, 1999). Sample items included “I plan to take more math classes than I have to in high school,” “I plan to complete all of my math homework on time,” and “I plan to participate in school related activities about math (like competitions or projects).” The items had acceptable internal consistency (α = 0.749) and were averaged so that higher values represented greater math education behavioral intentions.

Children's math attitudes were measured by 5 items rated on a scale from 1 (*Very Strongly Disagree*) to 6 (*Very Strongly Agree*; adapted from Sparks et al., 1997; Butler, 1999). Items included “I will use math a lot when I grow up”, “I enjoy studying math,” and “I think math is boring” (reverse-scored). The items had acceptable internal consistency (α = 0.761) and were reverse scored and averaged so that higher values represented more positive math attitudes.

Finally, children's math devaluing (Major and Schmader, 1998) was assessed by 5 items rated on a scale from 1 (*Very Strongly Disagree*) to 6 (*Very Strongly Agree*). Sample items included “I always feel good about myself when I do well on a math test” (reverse-scored) and “Doing well on math tests is very important to me” (reverse-scored). The items had acceptable internal consistency (α = 0.770) and were reverse-scored and averaged so that higher values represented greater math devaluing.

#### Procedure

Institutional Review Board approval was obtained from California State Polytechnic University, Pomona and permission was granted by the District Superintendent, each school principal, and each participating school teacher. Parents provided consent forms indicating whether their child could participate, and also signed a consent form if they participated in the questionnaire. Finally, students provided consent/assent forms in class if they chose to participate.

Researchers visited the classroom during the designated period and distributed the questionnaires, which were available in both English and Spanish. After students completed the questionnaires, they were each given a packet containing a consent form and questionnaire to take home to give to one of their parents. Completed parent questionnaires were typically mailed back to the researchers in a pre-paid enveloped or returned to the teacher and later collected by the researchers.

#### Analysis strategy

Although all of the relationships of interest exist at the student and parent level, the data came from an inherently hierarchical structure of children/parents (Level 1) nested within classrooms (Level 2) with different teachers, nested within different schools (Level 3). This sort of hierarchical structure often results in correlations of residuals among nested units that can bias the outcome of an ordinary least squares (OLS) regression by underestimating standard errors (Snijders and Bosker, 2011). Multilevel modeling (Raudenbush and Bryk, 2002) offers an appropriate remedy for analyzing nested data and is able to accommodate a wide range of data structures, including circumstances where the focal variables of interest are all situated on one level and the clustering is only a nuisance that prevents the use of OLS regression. To that end, two-level random intercept models were computed using the restricted maximum likelihood estimation, which adjusts for unequal sample sizes and is ideal for smaller datasets, with variance components estimator in SPSS Mixed Models Version 21 (IBM Corp., 2012). We were not able to use a three-level model because the number of schools (*N* = 8) was too small for a cluster analysis. However, the number of teachers (*N* = 24) was adequate for a two-level model to capture the nested nature of the data.

In addition, the data are also dyadic and non-independent as parents of the children also completed the questionnaire. The data were organized using the standard dyadic design and analyses are computed within dyad rather than between individuals (Kenny et al., 2006). For dyadic data, the slopes (the effects of predictors on Y for each dyad) are fixed to be equal across all dyads (Kenny et al., 2006). Instead the data are modeled through variation in the intercept at the Level 2 variable (teacher) across dyads. Finally, the Satterthwaite approximation was used to calculate degrees of freedom (Kenny et al., 2002).

For each model, the student's grade level and type of math class (honors or standard) were treated as control variables. Dyad type, child's math anxiety, and parent's math anxiety were entered into the model as main effects. Dyad type was effects coded as 1, 0.5, −0.5, and −1 for Father-Son, Father-Daughter, Mother-Son, and Mother-Daughter, respectively. To facilitate interpretation, child and parent math anxiety were group mean centered. All possible two-way interactions between the three variables were also entered into the model, as was a three-way interaction between dyad type, child's math anxiety, and parent's math anxiety. Significant interactions were graphed depicting the continuous variables (child and parent math anxiety) at one standard deviation above, at the mean, and one standard deviation below the mean (Aiken and West, 1991). Results for non-significant analyses are reported in the Supplementary Materials.

### Results

Descriptive statistics for all study variables are provided in Table 2 and the correlation matrix is displayed in Table 3. None of the variables were skewed or kurtotic to the extent that transformations were required.

**Table 2:** **Descriptive statistics of study variables**.

	***M***	***SD***	**Range**	**Skew**	**Kurtosis**
Child math anxiety	2.932	1.039	1–6	0.037	−0.233
Parent math anxiety	3.124	0.929	1–6	−0.048	0.070
Math self-efficacy	4.523	1.107	1–6	−0.752	0.077
Math GPA	2.267	1.204	0–4.30	−0.213	−0.824
Math behavioral intentions	4.471	0.801	1.33–6	−0.350	0.387
Math attitudes	4.384	0.973	1–6	−0.324	−0.076
Math devaluing	2.032	0.816	1–5.60	0.742	0.572

*For Child and Parent Math Anxiety, Math Behavioral Intentions, Math Attitudes, and Math Devaluing, 1 = Very Strongly Disagree and 6 = Very Strongly Agree. For Math Self-Efficacy, 1 = Not at all Confident and 6 = Very Confident*.

**Table 3:** **Correlations among continuous variables**.

	**1**	**2**	**3**	**4**	**5**	**6**	**7**
1. Child math anxiety	–						
2. Parent math anxiety	0.500^***^	–					
3. Math self-efficacy	−0.415^***^	−0.250^***^	–				
4. Math GPA	−0.371^***^	−0.210^***^	0.526^***^	–			
5. Math intentions	−0.200^***^	−0.050	0.416^***^	0.141^***^	–		
6. Math attitudes	−0.409^***^	−0.203^***^	0.441^***^	0.241^***^	0.483^***^	–	
7. Math devaluing	0.196^***^	0.099^*^	−0.379^***^	−0.134^***^	−0.598^***^	−0.486^***^	–

* *p < 0.05*,

*** *p < 0.001*.

#### Math self-efficacy

**Figure 1:**
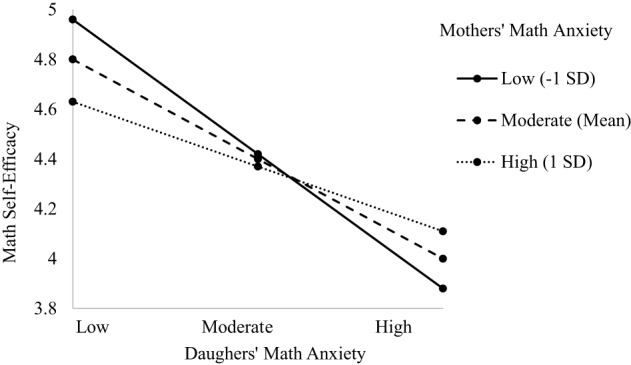
**Three-way interaction predicting math self-efficacy from Mother's and Daughter's math anxiety**.

The model predicting child's math self-efficacy indicated main effects of grade level, dyad type, and child's math anxiety, as well as the predicted three-way interaction between dyad type, child's math anxiety, and parent's math anxiety [*F*_(3, 577.13)_ = 3.311, *p* = 0.020; see Table 4]. The main effect of grade level indicated sixth grade students had higher math self-efficacy than seventh or eighth grade students (β = 0.291, *p* = 0.051); however, this effect was marginal and not of theoretical interest. The main effect of dyad type showed marginally lower math self-efficacy in the Mother-Daughter dyad compared to the other dyads (β = −0.312, *p* = 0.062). The main effect of child's math anxiety indicated a negative relationship such that greater math anxiety is associated with lower math self-efficacy (β = −0.472, *p* = 0.003), supporting Hypothesis 1a. However, both these main effects are subsumed in the three-way interaction. The three-way interaction model showed a significant relationship only for the Mother-Daughter dyad, supporting hypothesis 3a (β = 0.379, *p* = 0.037; see Figure 1). Daughters with lower math anxiety had higher math self-efficacy than daughters with higher math anxiety, and this relationship was stronger at lower levels of mother's anxiety (*b* = −0.512, *p* = 0.001), supporting Hypothesis 2 (Moderate mother anxiety: b = −0.380, *p* = 0.001; Higher mother anxiety: b = −0.248, *p* = 0.006). However, daughters with higher math anxiety had lower math self-efficacy when mothers had lower math anxiety, contrary to Hypothesis 2. There were no three-way interactions for other Parent-Child dyads (see Supplementary Results).

**Table 4:** **Three-way interaction predicting math self-efficacy and math GPA**.

	**Math self-efficacy**			**Math GPA**		
	***B***	**SE**	***P***	***B***	***SE***	***P***
Intercept	4.751	0.185	0.001	2.126	0.282	0.001
Class type	−0.167	0.099	0.093	0.415	0.112	0.001
Grade level	−0.167	0.078	0.041	−0.201	0.145	0.178
Dyad type	0.106	0.048	0.029	−0.007	0.050	0.883
Child math anxiety	−0.472	0.156	0.003	0.582	0.160	0.001
Parent math anxiety	−0.300	0.165	0.069	−0.165	0.183	0.367
Parent × Child Math anxiety	−0.233	0.171	0.175	−0.395	0.196	0.044
Dyad type × Child math anxiety	−0.056	0.050	0.262	−0.074	0.052	0.155
Dyad type × Parent math anxiety	−0.076	0.054	0.162	−0.003	0.058	0.960
Three-way interaction	−0.140	0.049	0.005	−0.108	0.054	0.048

#### Math class GPA

**Figure 2:**
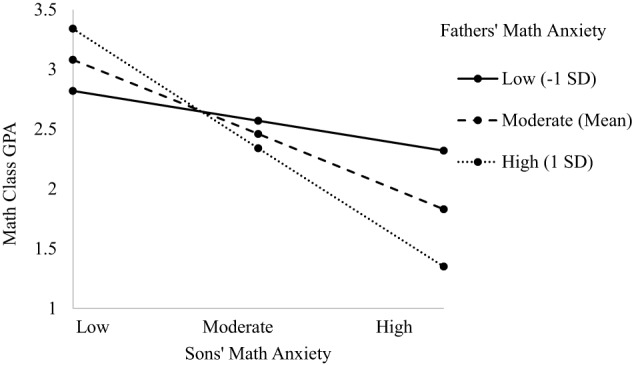
**Three-way interaction predicting math GPA from Father's and Son's math anxiety**.

The model predicting child's math class GPA indicated main effects of class type and child's math anxiety, a two-way interaction between parent and child math anxiety, as well as the predicted three-way interaction between dyad type, child's math anxiety, and parent's math anxiety [*F*_(3, 544.56)_ = 3.940, *p* = 0.048; see Table 4]. The main effect of class type indicated honors math students had higher math GPAs than standard math students (β = 0.415, *p* = 0.001). The main effect of child's math anxiety indicated a negative relationship such that greater math anxiety is associated with lower math GPAs (β = −0.582, *p* = 0.001), supporting Hypothesis 1b. The two-way interaction showed a negative relationship between the interaction of parent and child math anxiety and child's math GPA. When parents' math anxiety was lower, children with lower math anxiety had higher GPAs than children with higher anxiety (*b* = −0.442, *p* = 0.001). The same relationship held for parents with moderate (*b* = −0.422, *p* = 0.001) and higher (*b* = −0.402, *p* = 0.001) math anxiety, though the slopes are slightly lower as parent anxiety increases. The three-way interaction model showed a significant relationship only for the Father-Son dyad, supporting Hypothesis 3 (β = −0.457, *p* = 0.026; see Figure 2). When sons had lower math anxiety, their GPAs were higher compared to sons with higher math anxiety. Interestingly this relationship was strongest when fathers' had higher math anxiety, contrary to Hypothesis 2 (GPA around 3.5; *b* = −1.01, *p* = 0.005). There was a similar pattern when father's math anxiety was moderate (GPA around 3.2; *b* = −0.629, *p* = 0.008). The relationship was not significant when father's anxiety was lower. There were no three-way interactions for other Parent-Child dyads (see Supplementary Results).

#### Math behavioral intentions

**Figure 3:**
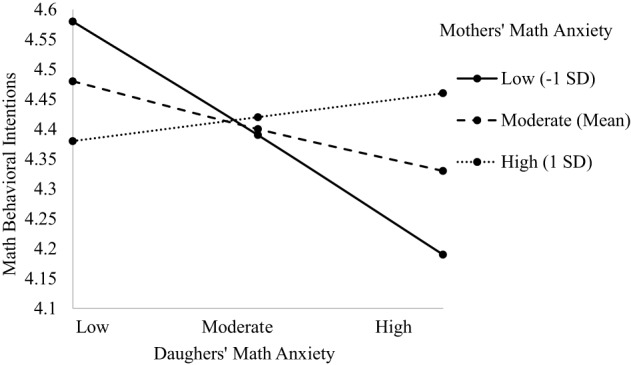
**Two-way interaction predicting math behavioral intentions from Mother's and Daughter's math anxiety**.

The model predicting child's math behavioral intentions indicated main effects of class type and child's math anxiety, and a two-way interaction between dyad type and child's math anxiety, [*F*_(3, 574.65)_ = 5.370, *p* = 0.021; see Table 5]. The predicted three-way interaction was not significant. The main effect of class type indicated honors math students had lower math behavioral intentions than standard math students (β = −0.158, *p* = 0.049). The main effect of child's math anxiety indicated a negative relationship such that greater math anxiety is associated with lower math behavioral intentions (β = −0.319, *p* = 0.008), supporting Hypothesis 1c. The two-way interaction model showed a marginally significant relationship only for the Mother-Daughter dyad that indicated the lower daughters' math anxiety, the higher their math behavioral intentions particularly if their mothers had lower math anxiety (β = 0.251, *p* = 0.054, see Figure 3), providing some support for Hypotheses 2 and 3a. However, when daughters had higher math anxiety, their math behavioral intentions were lower, particularly when mothers' math anxiety was low, contrary to Hypothesis 2. There were no two-way interactions for other Parent-Child dyads (see Supplementary Results).

**Table 5:** **Interactions predicting math behavioral intentions and math attitudes**.

	**Math behavioral intentions**			**Math attitudes**		
	***B***	***SE***	***P***	***B***	***SE***	***P***
Intercept	4.484	0.164	0.001	4.58	0.177	0.001
Class type	−0.158	0.080	0.049	−0.059	0.089	0.509
Grade level	−0.127	0.072	0.091	−0.259	0.072	0.002
Dyad type	0.061	0.037	0.097	0.195	0.042	0.001
Child math anxiety	−0.319	0.119	0.008	−0.467	0.134	0.001
Parent math anxiety	0.121	0.126	0.338	0.170	0.141	0.228
Parent × Child math anxiety	−0.021	0.131	0.870	−0.355	0.147	0.016
Dyad type × Child math anxiety	−0.088	0.038	0.021	−0.033	0.043	0.435
Dyad type × Parent math anxiety	0.032	0.042	0.438	0.054	0.047	0.247
Three-way interaction	−0.040	0.038	0.287	−0.135	0.043	0.002

#### Math attitudes

**Figure 4:**
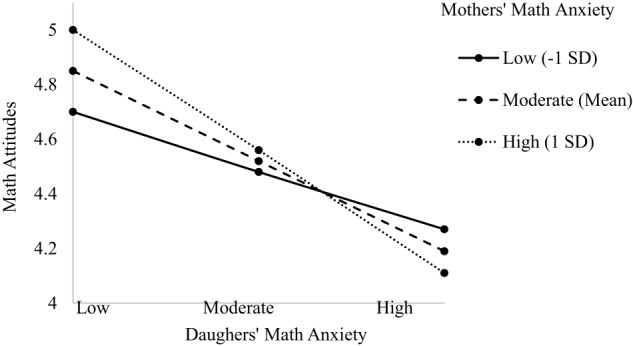
**Three-way interaction predicting math attitudes from Mothers' and Daughters' math anxiety**.

**Figure 5:**
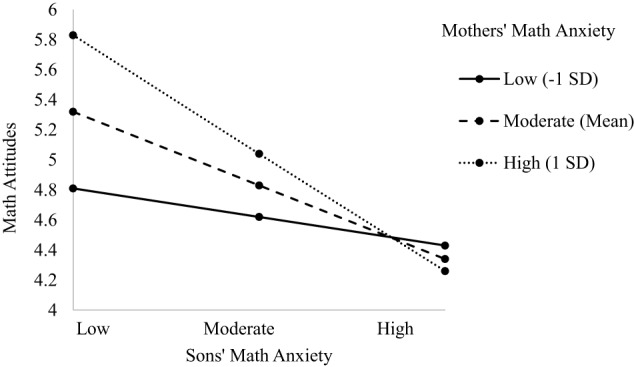
**Three-way interaction predicting math attitudes from Mothers' and Sons' math anxiety**.

**Figure 6:**
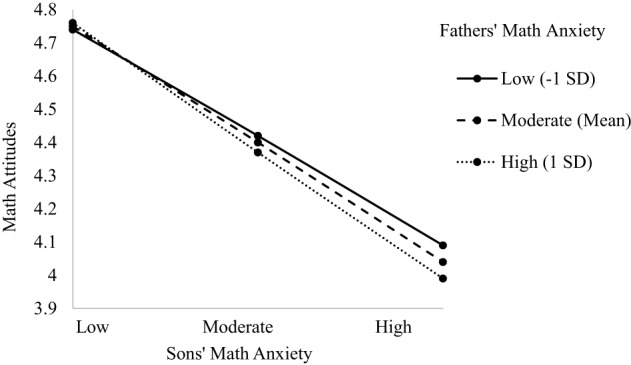
**Three-way interaction predicting math attitudes from Fathers' and Sons' math anxiety**.

The model predicting child's math attitudes indicated main effects of grade level, dyad type, and child's math anxiety (see Table 5). There was a two-way interaction between parent and child math anxiety, as well as a three-way interaction between dyad type, child's math anxiety, and parent's math anxiety, [*F*_(3, 572.83)_ = 4.580, *p* = 0.004; see Table 5]. The main effect of grade level indicated math attitudes become more negative as children progress through middle school (β = −0.259, *p* = 0.002; 6th grade *M* = 4.670, *SD* = 0.430; 7th grade *M* = 4.460, *SD* = 0.429; 8th grade *M* = 4.090, *SD* = 0.462). The main effect of dyad type indicated significant differences between dyads such that son's in Father-Son dyads had significantly more positive math attitudes (*M* = 4.690, *SD* = 0.530) than sons and daughters in all other dyad types (*M*s = 4.240−4.470, *SD*s = 0.427–0.510). This main effect is further explained by the three-way interaction and is described in more detail next. The main effect of child's math anxiety indicated a negative relationship such that greater math anxiety is associated with more negative math attitudes (β = −0.467, *p* = 0.001), consistent with Hypothesis 1d. The two-way interaction showed a negative relationship between the interaction of parent and child math anxiety and child's math attitudes (β = −0.355, *p* = 0.016). When children had lower math anxiety, their math attitudes were positive regardless of parent's math anxiety. However, when children's math anxiety was moderate to high, the higher parent's math anxiety, the lower children's math attitudes, supporting Hypothesis 2. This two-way interaction is subsumed by the three three-way interaction and is described in more detail next. The three-way interactions were significant for Mother-Daughter (β = 0.453, *p* = 0.004), Mother-Son (β = −0.231, *p* = 0.005), and the Father-Son dyad (β = −0.453, *p* = 0.004; see Figures 4–6). For the Mother-Daughter dyad, daughters with lower math anxiety had more positive math attitudes than daughters with higher math anxiety, and this relationship was strongest when mothers had higher anxiety (*b* = −0.414, *p* = 0.001) contrary to hypothesis 2, followed by moderate (*b* = −0.339, *p* = 0.001), followed by lower anxiety (*b* = −0.264, *p* = 0.003). For the Mother-Son dyad, sons with lower math anxiety had more positive math attitudes than sons with higher math anxiety, and this relationship was strongest when mothers had high anxiety (*b* = −0.430, *p* = 0.001) contrary to Hypothesis 2, followed by moderate (*b* = −0.321, *p* = 0.001), followed by low anxiety (*b* = −0.212, *p* = 0.030). For the Father-Son dyad, sons with lower math anxiety had more positive math attitudes than sons with higher math anxiety, and this relationship was strongest when fathers had high anxiety (*b* = −0.796, *p* = 0.005), contrary to Hypothesis 2, followed by moderate (*b* = −0.494, *p* = 0.007). This relationship was not significant when fathers had lower anxiety (*b* = −0.193, *p* = 0.286). There were no three-way interactions for other Parent-Child dyads (see Supplementary Results).

#### Math devaluing

**Figure 7:**
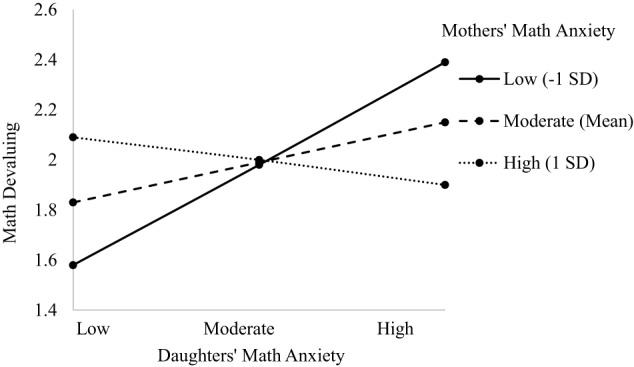
**Three-way interaction predicting math devaluing from Mothers' and Daughters' math anxiety**.

**Figure 8:**
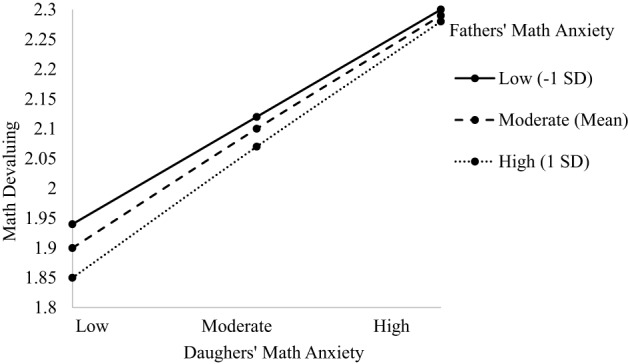
**Three-way interaction predicting math devaluing from Fathers' and Daughters' math anxiety**.

The model predicting child's math devaluing indicated main effects of grade level, dyad type, and child's math anxiety (see Table 6). There was a marginal three-way interaction between dyad type, child's math anxiety, and parent's math anxiety, [*F*_(3, 576.30)_ = 2.330; *p* = 0.074, see Table 6]. The main effect of grade level indicated math devaluing increases as children progress through middle school (β = 0.120, *p* = 0.040; 6th grade *M* = 1.880, *SD* = 0.224; 7th grade *M* = 2.040, *SD* = 0.220; 8th grade *M* = 2.150, *SD* = 0.206). The main effect of dyad type (β = −0.099, *p* = 0.011) indicated significant differences between dyads such that children in mother dyads had significantly more math devaluing (M-D: *M* = 2.070, *SD* = 0.184; M-S: *M* = 2.110, *SD* = 0.210), consistent with Hypothesis 3b, than children in father dyads (F-D: *M* = 1.880, *SD* = 0.314; F-S: *M* = 1.830, *SD* = 0.284). This main effect is further explained next by the marginal three-way interaction. The main effect of child's math anxiety indicated a positive relationship such that greater math anxiety is associated with more math devaluing (β = 0.253, *p* = 0.043), supporting Hypothesis 1e. The marginal three-way interaction showed that in the Mother-Daughter dyad, daughters with lower anxiety with mothers also lower in anxiety had less math devaluing than daughters with higher anxiety (*b* = 0.172, *p* = 0.015), providing some support for Hypothesis 2. However, daughters with higher math anxiety had greater math devaluing, particularly when mothers had lower math anxiety, contrary to Hypothesis 2. The relationship between daughters' anxiety and mothers' with moderate or high anxiety was not significant. The greater daughters' math anxiety, the more they devalued math; see Figures 7, 8). For the Father-Daughter dyad, fathers with lower math anxiety with daughters also low in anxiety had less math devaluing than daughters higher in anxiety (*b* = 0.429, *p* = 0.008), supporting Hypothesis 2b. The relationship between daughters' anxiety and fathers with moderate or high anxiety was not significant. The greater daughters' math anxiety, the more they devalued math. There were no three-way interactions for other Parent-Child dyads (see Supplementary Results).

**Table 6:** **Three-way interaction predicting math devaluing**.

	**Math devaluing**		
	***B***	***SE***	***p***
Intercept	1.846	0.146	0.001
Class type	0.003	0.079	0.968
Grade level	0.120	0.054	0.040
Dyad type	−0.099	0.039	0.011
Child math anxiety	0.253	0.125	0.043
Parent math anxiety	0.012	0.132	0.930
Parent × Child math anxiety	0.144	0.137	0.296
Dyad type × Child math anxiety	0.059	0.040	0.141
Dyad type × Parent math anxiety	−0.010	0.044	0.816
Three-way interaction	0.050	0.040	0.074

A summary of all the Study 1 three-way interaction results are summarized in Tables 7, 8.

**Table 7:** **Patterns of simple effects in significant three-way interactions for math self-efficacy and math GPA**.

	**Math self-efficacy**				**Math GPA**			
**Dyad type**		**H Par**	**M Par**	**L Par**				
Mother-Daughter	**H Chld**	Lower	Lower	^*^Lower	ns			
	**L Chld**	Higher	Higher	^*^Higher				
Mother-Son	ns				ns			
Father-Daughter	ns				ns			
Father-Son	ns					**H Par**	**M Par**	**L Par**
					**H Chld**	^*^Lower	^*^Lower	ns
					**L Chld**	^*^Higher	^*^Higher	ns

*H, High; M, Moderate; L, Low; Par, Parent; Chld, Child. Moderate is excluded for the child data because it reflects the mean and is more informative in graphic form*.

* *Reflects the stronger relationship in the interaction*.

**Table 8:** **Patterns of simple effects in significant three-way interactions for math attitudes and math devaluing**.

	**Math attitudes**				**Math devaluing**			
**Dyad type**		**H Par**	**M Par**	**L Par**		**H Par**	**M Par**	**L Par**
Mother-Daughter	**H Chld**	Lower^*^	Lower	Lower	**H Chld**	ns	ns	^*^Higher
	**L Chld**	Higher^*^	Higher	Higher	**L Chld**	ns	ns	^*^Lower
Mother-Son		**H Par**	**M Par**	**L Par**	ns			
	**H Chld**	Lower^*^	Lower	Lower				
	**L Chld**	Higher^*^	Higher	Higher				
Father-Daughter	ns					**H Par**	**M Par**	**L Par**
					**H Chld**	ns	ns	^*^Higher
					**L Chld**	ns	ns	^*^Lower
Father-Son		**H Par**	**M Par**	**L Par**	ns			
	**H Chld**	Lower^*^	Lower	Lower				
	**L Chld**	Higher^*^	Higher	Higher				

*H, High; M, Moderate; L, Low; Par, Parent; Chld, Child. Moderate is excluded for the child data because it reflects the mean and is more informative in graphic form*.

* *Reflects the stronger relationship in the interaction*.

### Discussion

The results generally support the prediction that parents' anxiety plays a role in children's math anxiety and the variables interact to predict several math education outcomes including math self-efficacy, math GPA, math behavioral intentions, math attitudes, and math devaluing. First, consistent with existing literature, there were five main effects (Hypotheses 1a–e) of child's math anxiety on all outcomes such that greater math anxiety was associated with lower math self-efficacy, lower math GPA, lower math behavioral intentions, more negative math attitudes, and greater math discounting. However, more interesting is the interaction of children's math anxiety with parents' math anxiety within gendered dyads. In support of Hypothesis 3, the same-gender parent-child dyads showed the most significant relationships, and more specifically in line with Hypothesis 3a, the Mother-Daughter dyad dominated the findings. The Mother-Daughter dyads' math anxiety predicted math self-efficacy, math behavioral intentions, math attitudes, and math devaluing, or four of the five hypothesized effects. The general pattern was consistent with the hypothesis that when both mothers' and daughters' math anxiety were low, daughters had more positive math outcomes compared to when mothers' and daughters' math anxiety were both high. For the cases when both mothers' and daughters' math anxiety were high, daughters had more negative math outcomes compared to when mothers' and daughters' math anxiety were both low for math attitudes. However, the simple effects for math self-efficacy, behavioral intentions, and math devaluing were contrary to Hypothesis 2 when daughters' math anxiety was high. In these cases, math outcomes were worst when mothers has lower math anxiety rather than higher math anxiety. A possible explanation is that mothers' involvement in daughters' math education, e.g., helping with homework, may moderate these unexpected findings (see Maloney et al., 2015). It seems that daughters' level of math anxiety was a better predictor of math self-efficacy, behavioral intentions, and math devaluing such that if daughters' math anxiety was high, math outcomes were negative even when mothers' math anxiety was lower. Perhaps the sample is mixed with mothers who are more active in helping their daughters with homework and others who are less active. This moderating variable may help explain the mixed results and should be measured in future studies that examine effects of gendered parent-child dyads.

The other same-gender parent-child dyad Father-Son showed two significant effects of the five possible. Interestingly, the Father-Son dyad was the only one to show a significant relationship to GPA. When both fathers and sons had lower math anxiety, math GPA was higher. Fortunately, even when fathers' math anxiety was higher, if sons had lower math anxiety, GPA was higher. Only when both fathers and sons had higher math anxiety was GPA lower.

Consistent with predictions there not many effects for mixed-gender parent-child dyads. For the Father-Daughter dyad, fathers with lower math anxiety with daughters also low in anxiety had less math devaluing than daughters higher in anxiety. For the Mother-Son dyad, sons with lower math anxiety had more positive math attitudes than sons with higher math anxiety, and this relationship was strongest when mothers had high anxiety. This is contrary to hypotheses and suggests that sons are showing reactance against mothers' higher math anxiety. Alternatively, this might suggest that transmission of parents' anxiety might not occur to the same extent in mixed-gender dyads. Also, mothers' involvement in sons' math education (e.g., math homework) may be a moderator. Further research is needed to investigate these gendered patterns.

In sum, this study contributes to the existing literature by helping to address several gaps. First, there is only one known published study that found effects of parents' math anxiety on children's math education outcomes, particularly their math performance. Second, this study examined these relationships using a gendered lens and found support for the gender stereotype literature that the transmission of math anxiety seems most prevalent among same-gender parent-child dyads, particular the Mother-Daughter dyad.

Since math anxiety is well-established as a correlate of many important math education outcomes, Study 2 sought to examine a second source of math anxiety, math-gender stereotypes. In addition, study 2 examines math anxiety as a potential mediator explaining the relationship between endorsement of math-gender stereotypes and math achievement.

## Study 2

The purpose of study 2 is two-fold: first to address a call for examination of antecedents of math anxiety (Ashcraft and Ridley, 2005; Jameson, 2014; Maloney et al., 2015) and second to address the call for a more mechanistic approach to examine mediators in the math anxiety and achievement domains (Gunderson et al., 2012).

### Gender stereotypes as a source of math anxiety

The Expectancy-Value Theory of achievement motivation describes the role of cultural and social factors that influence children's motivations, task values, expectations for success, and achievement related choices. In regards to mathematics achievement, the cultural milieu includes cultural stereotypes about math and gender roles appropriate in the math domain (Cheryan, 2012). Children are influenced by these cultural factors and also have their own perceptions of society's stereotypes about gender and math.

A great deal of the research using an expectancy-value framework work focuses on the transmission of math-gender stereotypes from parents to children and how stereotypes influence children's achievement outcomes. A logical extension of this work is that children's own endorsement of math-gender related stereotypes will affect their achievement outcomes. Indeed, research on stereotype threat shows that women who endorse stereotypes about women's inferiority in math are more susceptible to experiencing stereotype threat and subsequently show math performance decrements (Schmader et al., 2004). Anxiety is one mechanism through which stereotype threat negatively affects performance (Schmader et al., 2008). Stereotype threat is the fear of confirming a negative stereotype about one's social group (e.g., gender), and is particularly relevant in evaluative contexts (Steele and Aronson, 1995). Like math anxiety, stereotype threat also disrupts working memory capacity and depletes necessary cognitive resources to tackle complex problems (Schmader et al., 2008).

Taken together, the work on the expectancy-value model showing that parents' and teachers' gender stereotypes influence girls' math gender stereotypes, and work in stereotype threat showing stereotypes create anxiety that negatively impacts performance, the prediction of math-related gender stereotypes as a source of math anxiety seems logical.

Work by Beilock et al. (2010) found that math-anxious female teachers reinforced math-gender stereotypes and girls' endorsement of math-gender stereotypes mediated the effect of teacher anxiety on their math performance. Thus, teachers' anxiety affects gender math stereotypes not female students' anxiety, suggesting it works indirectly through stereotypes.

This finding provides further evidence that math-gender stereotypes can create math anxiety in girls.

### Math anxiety as a mechanism

Existing research on the negative effects of math anxiety suggests that it can function as a mechanism influencing math achievement. Studies have found that math anxiety lowers math performance regardless of actual math ability (Hembree, 1990; Maloney and Beilock, 2012; Park et al., 2014). Research by Chipman et al. (1992) found that math anxiety was a mediator in students' career choice. As Gunderson et al. (2012) stated, children's stereotypes, self-perceptions, math anxieties, and math achievement are all interconnected, therefore it is critical to know which component of children's gendered math attitudes is affected by specific behaviors from parents or teachers. One way to address this question is to test math-gender stereotypes as a predictor of math anxiety.

### Hypotheses

In Study 2 we argue that in addition to parents, math-related gender stereotypes are a source of math anxiety. We base this argument on research showing that math-gender stereotypes held by parents, teachers, and important others are linked to reports of higher math anxiety by females more so than males (Hembree, 1990). Further, students' endorsement of math-gender stereotypes mediated the effect of teacher anxiety on their math performance (Beilock et al., 2010). Additionally, we predict that math anxiety is a mechanism through which math-gender stereotypes negatively influence math outcomes for *both* girls and boys. Specifically we predict that (1) endorsement of math-gender stereotypes will predict math anxiety such that greater endorsement is associated with greater anxiety. Although we expect the variables to be correlated, and therefore bidirectional, we expect that (2) stereotypes are a stronger predictor of anxiety than vice versa. Further we predict that endorsement of math-gender stereotypes will be negatively related to (3a) math self-efficacy, (3b) math GPA, (3c) math behavioral intentions, (3d) math attitudes, (3e) and positively related to math devaluing. Finally, we predict that (4) math anxiety will fully mediate the relationship between math-gender stereotype endorsement and math outcomes. Although much of the focus on math-gender stereotypes has been on girls and women, (5) we predict these same relationships for boys, although the effects will be larger for girls. Boys who endorse math-gender stereotypes believe that boys and men *are* (descriptive stereotype) and *should be* (injunctive stereotype) superior to women in math. However, societal stereotypes of high math achievement reflects majority group members (Stephens et al., 2012), specifically males, Caucasians but also Asian Americans (Shih et al., 1999), and middle to upper middle class educated males. Not all males “benefit” from gender stereotypes about men's superiority math as we see in research on stereotype lift (Walton and Cohen, 2003). In our sample of racially diverse, low-income students, endorsing such stereotypes is likely to be threatening and remind male participants that they may not be high achieving in the math domain (Croizet and Claire, 1998).

### Method

#### Participants

The sample included 1342 students (*n* = 713, 53% female; *n* = 629, 47% male) from the same project described in Study 1; however, the sample included all students who completed a questionnaire in the classroom, regardless of whether their parent participated in the study. Students came from the same 8 schools in southern California and were enrolled in 6th grade (*n* = 361), 7th grade (*n* = 459), or 8th grade (*n* = 522) honors (*n* = 768), or standard (*n* = 574) math classes with one of 24 teachers. Students self-reported their race/ethnicity and the largest group represented was Latino/a or Hispanic (*n* = 910, 68%) followed by 131 (10%) multiracial, 111 (8%) Asian/Pacific Islander, and less than 5% each of remaining groups including Native American, White/Caucasian, and Other. Nearly 5% of students (*n* = 63) did not report a race or ethnicity. Students' ages ranged from 11 to 14 reflecting ages in the 6th through 8th grades.

#### Materials

Participants completed a 9-item measure of endorsement of math-related gender stereotypes on a scale from 1 (*Very Strongly Disagree*) to 6 (*Very Strongly Agree*). Sample items included “Girls are worse at math than boys,” “Girls are better at English, art, and history than math,” “Girls can do just as well as boys in math” (reverse-scored) and “Girls who like studying math are nerds.” The items had acceptable internal consistency (α = 0.803) and were reverse scored and averaged so that higher values represented more endorsement of math-related gender stereotypes.

Participants' math anxiety was assessed with the same measure from study 1. The items had acceptable internal consistency (α = 0.727) for this sample and were averaged so that higher values represented greater anxiety.

Participants' math self-efficacy was measured by the same 5-item measure reported in Study 1. The scale was internally consistent for this sample (α = 0.881) and items were averaged so that higher values represented greater math self-efficacy.

Participants' math education behavioral intentions were measured by the 6 items used in study 1. The items were reliable for this sample (α = 0.753) and items were averaged so that higher values represented greater math intentions.

Math attitudes were measure by the 5-item scale from study 1. The measure was reliable (α = 0.760) and averaged so that higher values indicated more positive attitudes.

Finally, participants' math devaluing was assessed by the 5 items used in study 1. The scale was internally consistent for this sample (α = 0.793) and items were averaged so that higher values represented greater math devaluing.

#### Procedure

The procedure was identical to Study 1. Participants completed the questionnaire during the assigned class time, which took 20–30 min, and the questionnaire was offered in both English and Spanish. The researchers collected the questionnaires and obtained the math class GPA roster from the teachers.

#### Analysis strategy

Like Study 1, the data came from an inherently hierarchical structure of children (Level 1) nested within classrooms with different teachers (Level 2). Multilevel modeling (MLM; Raudenbush and Bryk, 2002) was used to analyze the nested data, in this case in which the focal variables of interest are all situated on one level. Two-level random intercept models were computed using the restricted maximum likelihood estimation, which adjusts for unequal sample sizes and is ideal for smaller datasets, with variance components estimator in SPSS Mixed Models Version 21 (IBM Corp., 2012). Mediation analyses were conducted following guidelines by Baron and Kenny (1986) within a MLM framework to account for the nested data. Path A tested the relationship between the predictor variable (gender stereotypes) and the mediator (math anxiety), path B tested the relationship between the mediator and the outcome variable (math self-efficacy, math GPA, math intentions, math attitudes, and math devaluing), and path C tested the relationship between the predictor variable and the outcome variable. To test the significance of C' we computed the path from the predictor to the outcome variable while controlling for the mediator. In cases of significant mediation, reverse mediation will be tested and a Sobel test will be used to determine significant differences in the size of beta values. To examine gender differences and similarities in the mediation models, models will be run separately by gender. Consistent with Study 1, in all models we controlled for grade level and math class type.

### Results

For descriptive purposes, the means, standard deviations, and correlations for all study variables are provided in Table 9. All correlations are significant, except for one, and in the predicted direction, supporting Hypotheses 1 and 3a–e. There is a positive relationship between endorsement of math-gender stereotypes and math anxiety for both girls and boys. There is a negative relationship between math-gender stereotypes and math self-efficacy, math intentions, and math attitudes for both girls and boys and a negative relationship between math-gender stereotypes and math GPA for girls only. There is a marginal trend for boys, but it did not reach significance at the 0.05 level for a two-tailed test (*p* = 0.076). Math-gender stereotypes were positively correlated with math devaluing for both girls and boys. Math anxiety was negatively correlated with math self-efficacy, math GPA, math intentions, and math attitudes, and positively correlated with math devaluing for both girls and boys. Math self-efficacy was positively related to math GPA, math intentions, and math attitudes, and negatively related to math devaluing for both girls and boys. Math GPA was positively related to math intentions and attitudes, and negatively related to math devaluing for both girls and boys. Math intentions and math attitudes were positively related and both were negatively related to math devaluing for both gender groups.

**Table 9:** **Descriptive statistics and correlation matrix by gender for all variables**.

	***M***	***SD***	**1**	**2**	**3**	**4**	**5**	**6**	**7**
1. Gender stereotypes	2.318	0.756	–	0.191^***^	−0.086^*^	−0.116^**^	−0.193^***^	−0.252^***^	0.287^***^
	2.567	0.861							
2. Math anxiety	3.051	1.068	0.180^***^	–	−0.365^***^	−0.385^***^	−0.154^***^	−0.367^***^	0.133^***^
	2.800	1.048							
3. Math self-efficacy	4.469	1.107	−0.106^**^	−0.351^***^	–	0.520^***^	0.369^***^	0.392^***^	−0.350^***^
	4.436	1.163							
4. Math GPA	2.369	1.156	−0.074^∧^	−0.372^***^	0.550^***^	–	0.142^***^	0.231^***^	−0.183^***^
	2.088	1.228							
5. Math. intentions	4.511	0.808	−0.088*	−0.167^***^	0.440^***^	0.169^***^	–	0.504^***^	−0.584^***^
	4.381	0.829							
6. Matk attitudes	4.356	0.950	−0.156^***^	−0.336^***^	0.475^***^	0.305^***^	0.591^***^	–	−0.496^***^
	4.419	1.00							
7. Math devaluing	2.025	0.843	0.176^***^	0.182^***^	−0.383^***^	−0.157^***^	−0.626^***^	−0.594^***^	–
	2.089	0.891							

*Girls' values, are above the diagonal in bold and boys' values are below the diagonal in standard typeface*.

∧ *p = 0.076*,

* *p < 0.05*,

** *p < 0.01*,

*** *p < 0.001*.

#### Mediational models for girls

##### Math self-efficacy

The mediational models supported Hypothesis 4 that math anxiety mediates the effect of math-gender stereotypes on math self-efficacy, and the size of these relationship did not differ by gender, counter to Hypothesis 5. In support of the mediation models for girls, the more girls endorsed math-gender stereotypes, the greater their math anxiety (β = 0.257, *p* = 0.001; Path A, see Table 10A). Math anxiety was negatively related to math self-efficacy such that greater math anxiety predicted lower math self-efficacy (β = −0.382, *p* = 0.001; Path B, see Table 10B). The direct effect of math-gender stereotypes on math self-efficacy was negative, such that greater endorsement of stereotypes predicted lower math self-efficacy (β = −0.125, *p* = 0.024; Path C, see Table 10B). However, when math anxiety was entered into the model the effect of math-gender stereotypes on math self-efficacy was no longer significant (Path C', see Table 10B). Thus, math anxiety is a mediator of the relationship between math-gender stereotypes and math self-efficacy, supporting Hypothesis 4. Since the data are correlational and cross-sectional, a reverse mediation analysis was computed to rule out math-gender stereotypes as a mediator, particularly because the variables are correlated, *r*_(696)_ = 0.191, *p* = 0.001. The reverse mediation model showed the same pattern of relationships for math anxiety predicting gender stereotypes, although it was weaker (β = 0.133, *p* = 0.001). A sobel test confirmed that the beta for math-gender stereotypes predicting anxiety (β = 0.257) was significantly greater than the beta for anxiety predicting stereotypes (β = 0.133), *z* = 2.120, *p* = 0.034, supporting Hypothesis 2. Math-gender stereotypes also predicted self-efficacy (β = −0.123, *p* = 0.026), which was also weaker than the standard mediational model, z = 3.910, *p* = 0.001. However, when math-gender stereotypes was entered as the mediator, the direct relationship between math anxiety and self-efficacy did not change (C path β = −0.382, *p* = 0.001; C' path β = −0.381, *p* = 0.001), indicating math-gender stereotypes does not mediate the relationship between math anxiety and math self-efficacy, supporting Hypothesis 4.

Table 10**Mediation model statistics for girls' math self-efficacy arid math GPA**.

**Table:** 

**A**	**Outcome: Math anxiety (M)**			
**Antecedent**	**Identifier**	**Coeff**	***SE***	***P***
Intercept	i_1_	2.650	0.180	0.001
Grade level	Control	0.184	0.080	0.029
Class type	Control	−0.059	0.096	0.542
Math Anxiety (M)	–	–	–	–
Gender Stereotypes (X)	A	0.257	0.052	0.001
Model Summary	*F*_(1, 692)_ = 24.289, *p* = 0.001

**Table:** 

**B**	**Outcome: Math Self-Efficacy (Y)**				**Outcome: Math GPA (Y)**			
**Antecedent**	**Identifier**	**Coeff**	***SE***	***P***	**Identifier**	**Coeff**	***SE***	***P***
Intercept	i_2_	5.734	0.199	0.001	i_2_	3.400	0.278	0.001
Grade level	Control	−0.032	0.073	0.680	Control	−0.102	0.145	0.488
Class type	Control	−0.127	0.095	0.182	Control	0.320	0.097	0.001
Math Anxiety (M)	b	−0.382	0.037	0.001	b	−0.325	0.096	0.001
Gender Stereotypes (X)	c	−0.125	0.055	0.024	c	−0.137	0.055	0.012
	c′	−0.034	0.053	0.524	c′	−0.043	0.052	0.406
Model summary	*F*_(1, 690)_ = 101.69, *p* = 0.001				*F*_(1, 639)_ = 106.60, *p* = 0.001			

##### Math GPA

The mediational models support Hypothesis 4 that math anxiety mediates the effect of math gender stereotypes on math GPA, and that the size of these relationship differs by gender, supporting Hypothesis 5. In support of the mediation models for girls, math anxiety was negatively related to math GPA such that greater math anxiety predicted lower math GPA (β = −0.325, *p* = 0.001; Path B, see Table 10B). The direct effect of gender stereotypes on math GPA was negative, such that greater endorsement of math-gender stereotypes predicted lower math GPA (β = −0.137, *p* = 0.012; Path C, see Table 10B). However, when math anxiety was entered into the model the effect of math-gender stereotypes on math GPA was no longer significant. Thus, math anxiety is a mediator for the relationship between math-gender stereotypes and math GPA. The same reverse mediation analysis was conducted and indicated the direct effect of anxiety on math GPA did not significantly change when math-gender stereotypes was added to the model, thus math anxiety is the mediator rather than math-gender stereotypes.

##### Math intentions

The mediational models testing math anxiety as a mediator of the effect of math-gender stereotypes on math intentions was not significant, contrary to Hypothesis 4; however the pattern of relationships were in the predicted directions (see Table 11A). For girls, math anxiety was negatively related to math intentions such that greater math anxiety predicted lower math intentions (β = −0.108, *p* = 0.001; Path B, see Table 11B). The direct effect of math-gender stereotypes on math intentions was negative, such that greater endorsement of stereotypes predicted lower math intentions (β = −0.205, *p* = 0.012; Path C, see Table 11B). However, when math anxiety was entered into the model the effect of math-gender stereotypes on math intentions was weaker (β = −0.187, *p* = 0.001; Path C', see Table 11B), but the difference was not statistically significant, z = 0.322, *p* > 0.05. This indicates that math anxiety does not mediate the relationship between math-gender stereotypes and math intentions.

Table 11**Mediation model statistics for girls' math intentions, attitudes, and devaluing**.

**Table:** 

**A**	**Outcome: Math Anxiety (M)**			
**Antecedent**	**Identifier**	**Coeff**	***SE***	***P***
Intercept	i_1_	2.650	0.180	0.001
Grade level	Control	0.184	0.080	0.029
Class type	Control	−0.059	0.096	0.542
Math anxiety (M)	–	–	–	–
Gender stereotypes (X)	a	0.257	0.052	0.001
Model summary	*F*_(1, 692)_ = 24.289, *p* = 0.001

**Table:** 

**B**	**Outcome: Math intentions (Y)**				**Outcome: Math Attitudes (Y)**			
**Antecedent**	**Identifier**	**Coeff**	**SE**	***p***	**Identifier**	**Coeff**	**SE**	***P***
Intercept	i_2_	4.760	0.132	0.001	i_2_	5.119	0.145	0.001
Grade level	Control	0.164	0.116	0.178	Control	−0.161	0.062	0.015
Class type	Control	−0.035	0.073	0.630	Control	0.021	0.080	0.794
Math anxiety (M)	b	−0.108	0.028	0.001	b	−0.309	0.031	0.001
Gender stereotypes (X)	c	−0.205	0.039	0.001	c	−0.314	0.045	0.001
	c'	−0.187	0.040	0.001	c'	−0.248	0.044	0.001
Model summary	*F*_(1, 689)_ = 8.857, *p* = 0.001				*F*_(1, 691)_ = 33.651, *p* = 0.001

**Table:** 

**C**	**Outcome: Math Devaluing (Y)**			
**Antecedent**	**Identifier**	**Coeff**	***SE***	***p***
Intercept	i_2_	0.826	0.377	0.043
Grade level	control	0.131	0.054	0.029
Class type	control	−0.052	0.074	0.478
Math anxiety (M)	b	0.098	0.030	0.001
Gender stereotypes (X)	c	0.322	0.040	0.001
	c′	0.313	0.041	0.001
Model summary	*F*_(1, 691)_ = 15.967, *p* = 0.001			

##### Math attitudes

The mediational models testing math anxiety as a mediator of the effect of math-gender stereotypes on math attitudes was not significant, contrary to Hypothesis 4; however, the pattern of relationships were in the predicted directions. For girls, math anxiety was negatively related to math attitudes such that greater math anxiety predicted lower math attitudes (β = −0.309, *p* = 0.001; Path B, see Table 11B). The direct effect of math-gender stereotypes on math attitudes was negative, such that greater endorsement of stereotypes predicted lower math attitudes (β = −0.314, *p* = 0.001; Path C, see Table 11B). However, when math anxiety was entered into the model the effect of math-gender stereotypes on math attitudes was weaker (β = −0.248, *p* = 0.001; Path C', see Table 11B), but the difference was not statistically significant, z = 1.049, *p* > 0.05. This indicates that math anxiety does not mediate the relationship between math-gender stereotypes and math attitudes.

##### Math devaluing

The mediational models testing math anxiety as a mediator of the effect of math-gender stereotypes on math devaluing was not significant, contrary to Hypothesis 4; however the pattern of relationships were in the predicted directions. For girls, math anxiety was positively related to math devaluing such that greater math anxiety predicted greater math devaluing (β = 0.098, *p* = 0.001; Path B, see Table 11C). The direct effect of math-gender stereotypes on math devaluing was positive, such that greater endorsement of stereotypes predicted greater math devaluing (β = 0.322, *p* = 0.001; Path C, see Table 11C). However, when math anxiety was entered into the model the effect of math-gender stereotypes on math devaluing was weaker (β = 0.313, *p* = 0.001; Path C', see Table 11C), but the difference was not statistically significant, z = 0.122, *p* > 0.05. This indicates that math anxiety does not mediate the relationship between math-gender stereotypes and math devaluing.

#### Mediational models for boys

##### Math self-efficacy

In support of Hypothesis 4, the mediational model for math self-efficacy was significant. In support of the mediation models for boys, the more boys endorsed math-gender stereotypes, the greater their math anxiety (β = 0.214, *p* = 0.001; Path A, see Table 12A). Math anxiety was negatively related to math self-efficacy such that greater math anxiety predicted lower math self-efficacy (β = −0.383, *p* = 0.001; Path B, see Table 12B). The direct effect of math-gender stereotypes on math self-efficacy was negative, such that greater endorsement of stereotypes predicted lower math self-efficacy (β = −0.128, *p* = 0.017; Path C, see Table 12B). However, when math anxiety was entered into the model the effect of math-gender stereotypes on math self-efficacy was no longer significant. Thus, math anxiety is a mediator of the relationship between math-gender stereotypes and math self-efficacy. Reverse mediation was not significant, indicating math-gender stereotypes is not a mediator of the relationship between math anxiety and math self-efficacy.

Table 12**Mediation model statistics for boys' math self-efficacy and Math GPA**.

**Table:** 

**A**	**Outcome: Math anxiety (M)**			
**Antecedent**	**Identifier**	**Coeff**	**SE**	***p***
Intercept	i_1_	2.283	0.185	0.001
Grade level	control	−0.010	0.086	0.912
Class type	control	−0.070	0.099	0.477
Math anxiety (M)	–	–	–	–
Gender stereotypes (X)	A	0.214	0.048	0.001
Model summary	*F*_(1, 613)_ = 19.861, *p* = 0.001

**Table:** 

**B**	**Outcome: Math self-efficacy (Y)**				**Outcome: Math GPA (Y)**			
**Antecedent**	**Identifier**	**Coeff**	***SE***	***P***	**Identifier**	**Coeff**	***SE***	***p***
Intercept	i_2_	5.518	0.222	0.001	i_2_	3.05	0.261	0.001
Grade level	Control	−0.184	0.010	0.081	Control	−0.230	0.129	0.089
Class type	Control	−0.118	0.105	0.258	Control	0.244	0.108	0.023
Math anxiety (M)	b	−0.383	0.041	0.001	b	−0.405	0.043	0.001
Gender stereotypes (X)	c	−0.128	0.054	0.017	c	−0.092	0.056	0.101
	c′	−0.051	0.051	0.323	c′	−0.006	0.053	0.910
Model summary	*F*_(1, 612)_ = 74.888, *p* = 0.001				*F*_(1, 564)_ = 80.958, *p* = 0.001			

##### Math GPA

In partial support of Hypothesis 4, there was an indirect effect of math-gender stereotypes on math GPA; however, the mediational model was not significant due to the marginal trend between math-gender stereotypes and math GPA for boys. The model paths showed the same pattern, that math-gender stereotypes was positively related to math anxiety, and math anxiety was negatively related to math GPA such that greater math anxiety predicted lower math GPA (β = −0.405, *p* = 0.001; Path B, see Table 12B). The direct effect of math-gender stereotypes on math GPA was negative but marginal (β = −0.092, *p* = 0.101); however, accounting for math anxiety reduced this effect substantially (β = −0.006, *p* = 0.91; Path C, see Table 12B), indicating an indirect effect of gender stereotypes. A reverse mediation model is not plausible given the lack of a significant direct relationship between math-gender stereotypes and math GPA for boys. Since the mediational model for girls was significant but not for boys, this provides support for Hypothesis 5 that the size of the relationship differs by gender.

##### Math intentions

Contrary to Hypothesis 4, the mediational model for math intentions was not significant; however the pattern of relationships were in the predicted directions (see Table 13A). Math anxiety was negatively related to math intentions such that greater math anxiety predicted lower math intentions (β = −0.148, *p* = 0.001; Path B, see Table 13B). The direct effect of math-gender stereotypes on math intentions was negative, such that greater endorsement of stereotypes predicted lower math intentions (β = −0.096, *p* = 0.017; Path C, see Table 13B). When math anxiety was entered into the model the effect of math-gender stereotypes on math intentions remained significant (β = −0.082, *p* = 0.040; Path C', see Table 13B) and the reduction in the beta value was not significant, z = 0.0247, *p* > 0.05. Thus, math anxiety is a not a mediator of the relationship between math-gender stereotypes and math intentions.

Table 13**Mediation model statistics for boys' math intentions, attitudes, and devaluing**.

**Table:** 

**A**	**Outcome: Math anxiety (M)**			
**Antecedent**	**Identifier**	**Coeff**	***SE***	***p***
Intercept	i_1_	2.283	0.185	0.001
Grade level	Control	−0.010	0.086	0.912
Class type	Control	−0.070	0.099	0.477
Math anxiety (M)	–	–	–	–
Gender stereotypes (X)	a	0.214	0.048	0.001
Model summary	*F*_(1, 613)_ = 19.861, *p* = 0.001

**Table:** 

**B**	**Outcome: Math intentions (Y)**				**Outcome: Math attitudes (Y)**			
**Antecedent**	**Identifier**	**Coeff**	***SE***	***p***	**Identifier**	**Coeff**	***SE***	***p***
Intercept	i_2_	5.87	0.499	0.001	i_2_	6.73	0.475	0.001
Grade level	Control	−0.131	0.071	0.081	Control	−0.195	0.067	0.011
Class type	Control	−0.176	0.078	0.024	Control	−0.038	0.085	0.658
Math anxiety (M)	b	−0.148	0.031	0.001	b	−0.325	0.036	0.001
Gender stereotypes (X)	c	−0.096	0.040	0.017	c	−0.191	0.046	0.001
	c′	−0.082	0.040	0.040	c′	−0.134	0.044	0.002
Model summary	*F*_(1, 607)_ = 8.818, *p* = 0.001				*F*_(1, 612)_ = 20.241, *p* = 0.001

**Table:** 

**C**	**Outcome: Math devaluing (Y)**			
**Antecedent**	**Identifier**	**Coeff**	***SE***	***p***
Intercept	i_2_	0.653	0.571	0.267
Grade level	Control	0.142	0.081	0.096
Class type	Control	0.032	0.083	0.703
Math anxiety (M)	b	0.165	0.034	0.001
Gender stereotypes (X)	C	0.173	0.041	0.001
	c′	0.140	0.041	0.001
Model summary	*F*_(1, 612)_ = 8.657, *p* = 0.001			

##### Math attitudes

Contrary to Hypothesis 4, the mediational model for math attitudes was not significant; however the pattern of relationships were in the predicted directions. Math anxiety was negatively related to math attitudes such that greater math anxiety predicted lower math attitudes (β = −0.325, *p* = 0.001; Path B, see Table 13B). The direct effect of math-gender stereotypes on math attitudes was negative, such that greater endorsement of stereotypes predicted more negative math attitudes (β = −0.191, *p* = 0.007; Path C, see Table 13B). When math anxiety was entered into the model the effect of math-gender stereotypes on math attitudes remained significant (β = −0.134, *p* = 0.002; Path C', see Table 13B) and the reduction in the beta value was not significant, z = 0.895, *p* > 0.05. Thus, math anxiety is a not a mediator of the relationship between math-gender stereotypes and math attitudes.

##### Math devaluing

Contrary to Hypothesis 4, the mediational model for math devaluing was not significant; however the pattern of relationships were in the predicted directions. Math anxiety was positively related to math devaluing such that greater math anxiety predicted greater math devaluing (β = 0.165, *p* = 0.001; Path B, see Table 13C). The direct effect of math-gender stereotypes on math devaluing was positive, such that greater endorsement of stereotypes predicted greater math devaluing (β = 0.1731, *p* = 0.001; Path C, see Table 13C). When math anxiety was entered into the model the effect of math-gender stereotypes on math devaluing remained significant (β = 0.140, *p* = 0.001; Path C', see Table 13C) and the reduction in the beta value was not significant, z = 0.569, *p* > 0.05. Thus, math anxiety is a not a mediator of the relationship between math-gender stereotypes and math devaluing.

### Discussion

Results from Study 2 supported the hypotheses that endorsement of math-gender stereotypes was negatively related to two math outcomes including math self-efficacy and math GPA for both girls and boys. Math anxiety fully mediated the relationship between endorsement of math-gender stereotypes and math self-efficacy and math GPA. Thus, the persistence of math-based gender stereotypes in the US are not only inaccurate, but they are harmful for both girls' and boys' math achievement.

Results support the argument that endorsement of math-gender stereotypes may serve as an antecedent to math anxiety. Although the variables are correlated, regression analyses indicate the stronger relationship is from stereotypes to anxiety for two math outcomes: self-efficacy for girls and boys, and math GPA for girls. Further, mediational analyses indicate that math anxiety, not math gender stereotypes, mediate the relationship between endorsement of math-gender stereotypes and negative math achievement. Interestingly, although the predicted patterns of relationships emerged, math anxiety did not mediate the relationship between math-gender stereotypes and math attitudes, intentions, or devaluing. It may be that gender stereotypes have a stronger relationship with more achievement outcomes (e.g., self-efficacy and GPA) and math anxiety serves as a mediator of these relationships, but not for more attitudinal variables. Future research is needed to better understand the conditions under which math anxiety serves as a mediator between math-gender stereotypes and math outcomes.

This study provided initial evidence that the socialization of, and endorsement of math-gender stereotypes among girls and boys is related to negative math achievement. Further, math anxiety serves as a mechanism for lower math self-efficacy and math performance but not math attitude related variables.

## General discussion

The purpose of this research was to further probe the social determinants of adolescents' math anxiety by examining the relationship between same and other-gender parents' math anxiety with their child's math anxiety, and the downstream effects of math anxiety on math education outcomes. The first study addressed a gap in the literature by examining parents' own math anxieties (Gunderson et al., 2012; see Maloney et al., 2015). Results confirmed expectations that parents' anxiety is related to children's anxiety and these two variables interact to predict math education outcomes. In doing so, the first study also advances our knowledge of the gendered nature of the intergenerational transfer of math anxiety (Gniewosz and Noack, 2012). The results for mother-daughter dyads were mixed, supporting the hypothesis when daughters' and mothers' math anxiety were both low, but not consistently supporting hypotheses when daughters' and mothers' math anxiety were both high. Future research should further examine the mixed results by measuring possible moderating variables such as the extent to which mothers are involved in daughters' math education, such as helping with homework (see Maloney et al., 2015). It may be the case that for daughters whose mother does not help much, daughters' own math anxiety is a better predictor of math self-efficacy, behavioral intentions, and math devaluing. However, when mothers are actively involved in helping daughters with math homework, the predicted interaction of low-low and high-high parent-child math anxiety may reflect the hypothesized relationships.

Results from study 1 indicate that parents' anxiety plays a role in children's math anxiety and the variables interact to predict several math education outcomes including math self-efficacy, math GPA, math behavioral intentions, math attitudes, and math devaluing. Consistent with existing literature, children with greater math anxiety had lower math self-efficacy, lower math GPA, lower math behavioral intentions, more negative math attitudes, and greater math discounting. However, more interesting was the interaction of children's math anxiety with parents' math anxiety within gendered dyads. The same-gender parent-child dyads showed the most significant relationships, and more specifically the Mother-Daughter dyad dominated the findings. The Mother-Daughter dyads' math anxiety predicted math self-efficacy, math behavioral intentions, math attitudes, and math devaluing. The general pattern was consistent with the hypothesis that when both mothers' and daughters' math anxiety were low, daughters had more positive math outcomes compared to when mothers' and daughters' math anxiety were both high, with exceptions as discussed previously.

Interestingly, the Father-Son dyad was the only one to show a significant relationship to GPA. When both fathers and sons had lower math anxiety, math GPA was higher. Fortunately, even when fathers' math anxiety was higher, if sons had lower math anxiety, GPA was higher. Only when both fathers and sons had higher math anxiety was GPA lower. This finding is novel and should be replicated in future studies. Mothers' anxiety did not predict daughters' GPA, thus there may be other variables intervening in this relationship that are not present in the Father-Son dyad. This likely reflects the gendered nature of math stereotypes and the fact that girls and women are more negatively impacted by cultural biases.

Study 2 addressed a call for a mechanistic approach (Gunderson et al., 2012), and demonstrated that math anxiety is a mechanism through which math-gender stereotypes negatively influence performance related math outcomes for *both* girls and boys. Further, the results suggest that endorsement of math-gender stereotypes may be an antecedent for developing math anxiety for both boys and girls.

In sum, two studies contributed to the existing literature by helping to address several gaps. First, there is only one known published study that found effects of parents' math anxiety on children's math education outcomes, particularly their math performance (Maloney et al., 2015). Second, this study examined these relationships using a gendered lens and found support for the gender stereotype literature that the transmission of math anxiety seems most prevalent among same-gender parent-child dyads, particularly the Mother-Daughter dyad. Further, the results provided initial evidence that the socialization of, and endorsement of math-gender stereotypes among girls and boys is related to negative math achievement.

### Limitations

Although the studies make novel contributions to the literature, they are not without weaknesses. First, the data are correlational and cross-sectional. Longitudinal data over at least a full school year would be more informative regarding potential causal relationships. Although the mediational analyses for performance outcomes held after testing for reverse mediation, a stronger case for causality and direction of effects can be made with longitudinal data.

A drawback of Study 1 is that only one parent completed the questionnaire, limiting the full test of the gender of parent who might be most influential on daughters and sons. It can be argued that the parent completing the questionnaire may be the one most involved in the child's math education, but this assumption is tenuous. Further, the sample size of fathers was smaller, which perhaps made the analysis of Father-Daughter and Father-Son dyads underpowered. The fathers who did participated may be particularly good in math and therefore the results with fathers may not be representative of the full spectrum of Father-Daughter and Father-Son relationships regarding math education.

The response rate in Study 1 was 51%, thus the sample of parents who participated is likely different in some ways than parents who did not participate. Without data on non-participating parents, this is difficult to know. We do know that the children whose parents participated did vary in some systematic ways from children whose parents did not participate^2^. Further, the sample of parents reflects racial, ethnic, and national diversity. There may be cultural differences in norms regarding parental involvement in children's math education that are not captured in this study^3^.

### Implications

Despite these limitations the studies provide several contributions to the literature and the data can be used to inform school-based interventions. For example, stereotype busting interventions for teachers, parents, and students may be helpful. Given that several meta analyses show there is no longer a gender gap in math performance (Hyde et al., 2008; Lindberg et al., 2010), educators need to spread awareness to break down gender stereotypes as a barrier to math achievement. Further, anti-math anxiety training seems to be critical for math teachers and parents, particularly mothers. Such training can help boost math self-efficacy, which can be transmitted to students (Hendel and Davis, 1978; Tooke and Lindstrom, 1998; Gresham, 2007). Similarly, parents need to know about the subtle effects they may have on their children in communicating their own math anxiety. It is well known that parental involvement in students' education influences academic outcomes (Jeynes, 2007). Educational campaigns to promote parental involvement and educate parents on the importance of math education for all students might help encourage parents to support their children's math education endeavors. Specifically, educating parents on the impact that their beliefs and actions may have on their children's academic success would be of benefit.

Finally, schools should implement math anxiety reducing workshops for students. All students, girls and boys, will benefit from lower math anxiety. Perhaps what is ultimately needed is an overhaul of the US education system to focus less on competition and testing and more on collaboration and learning. Research has shown that when there is less focus on getting the right answers, and providing students with social support, students show less math anxiety (Turner et al., 2002). Also, when teachers emphasize incremental intelligence, working hard and making mistakes to learn, students have better academic achievement (Dweck, 2006).

## Author note

Bettina J. Casad, Department of Psychological Sciences, University of Missouri-St. Louis; Patricia Hale, Department of Mathematics and Statistics, California State Polytechnic University, Pomona; Faye L. Wachs, Department of Psychology and Sociology, California State Polytechnic University, Pomona. The work in this manuscript was supported by the National Science Foundation under Grant No. 0734124. Any opinions, findings, and conclusions or recommendations expressed in this paper are those of the authors and do not necessarily reflect the views of the National Science Foundation. The authors would like to thank Brandon Nakawaki for consultation on data analysis and members of the Cal Poly Pomona STEPS to Math Success research team for their assistance with data collection. Thank you to the school district administrators, teachers, parents, and students who participated and allowed us to conduct this study during school time.

### Conflict of interest statement

The authors declare that the research was conducted in the absence of any commercial or financial relationships that could be construed as a potential conflict of interest.

## Footnotes

^1^There were some marginal and significant differences in the characteristics and demographics of the students whose parent participated compared to students whose parent did not participate. Students without a participating parent had lower self-efficacy, and marginally greater endorsement of gender stereotypes and math. However, there were no significant differences for math anxiety, math attitudes, math devaluing, and math behavioral intentions. There was a marginal difference in the gender of the child such that females were more likely to have had a parent participate than males. There was a difference in the race of the child such that racial non-minority students, that is Caucasians or Asian Americans, were more likely to have had a parent participate than minority students. There were no differences for GPA, class type (honors/non honors) or grade level. See the Supplemental Results for the analyses.

^2^In the United States, grades in school reflect the level of one's education. Sixth grade is either the final year of elementary school education, or the first year of middle school education. Seventh and eighth grades are typically offered in middle schools, which is the educational period before high school where students earn their diploma. Honors math classes indicate the students are high achieving and are over-qualified for the math classes offered to most students. In the United States, students are often tracked into higher level math classes, such as ones to prepare them for college, and these courses are often called “honors” courses or advanced placement.

^3^We thank an anonymous reviewer for pointing this out.
