# Supplementary material for: Parent-child math anxiety and math-gender stereotypes predict adolescents' math education outcomes
Source: Front Psychol. 2015 Nov 3;6:1597. doi: 10.3389/fpsyg.2015.01597 (PMC4630312; doi:10.3389/fpsyg.2015.01597)
Supplement: Supplementary file 1 [file DataSheet1.DOCX]

**Study 1 Supplementary Results for Sample Differences for Participants With and Without Parental Data**

*Table 1*. Comparison Between Students With and without Dyadic Data on Study Variables

|  | **With Parental Data** | | **Without Parental Data** | | **Statistics** |
| --- | --- | --- | --- | --- | --- |
|  | *M* | *SD* | *M* | *SD* |  |
| **Outcomes** | | | | | |
| Child Math Anxiety | 2.931 | 1.039 | 2.933 | 1.086 | *t*(1340) = .036, *p* = .971 |
| Math Self-Efficacy | 4.522 | 1.107 | 4.398 | 1.152 | *t*(1358) = -2.009, *p* = .045 |
| Math GPA | 2.295 | 1.203 | 2.257 | 1.185 | *t*(1254) = -.564, *p* = .573 |
| Math Behavioral Intentions | 4.471 | .801 | 4.433 | .834 | *t*(1352) = -.855, *p* = .393 |
| Math Attitudes | 4.384 | .973 | 4.387 | .977 | *t*(1358) = .047, *p* = .963 |
| Math Devaluing | 2.032 | .033 | 2.074 | .904 | *t*(1356) = .887, *p* = .375 |
| Gender Stereotypes | 2.390 | .807 | 2.471 | .823 | *t*(1332) = 1.802, *p* = .072 |

*Table 2*. Comparison Between Students With and without Dyadic Data on Demographic Variables

| **Demographics** | **Statistics** |
| --- | --- |
| Grade Level | b = -.094, *p* = .207, χ^2^(4) = 8.328, *p* = .08 |
| Honors | b = .132, *p* = .626, χ^2^(4) = 8.328, *p* = .08 |
| Race | χ^2^(1) = 8.197, *p* = .005 |
| Gender | χ^2^(1) = 3.725, *p* = .054 |

**Study 1 Supplementary Results for Non-Significant Post-Hoc Analyses**

*Table 3*. Three-Way Interaction Post-Hoc Analyses for Math Self-Efficacy and Math GPA

Math Self-Efficacy Math GPA

b S.E. *p* b S.E. *p*

3-Way interaction -.140 .049 .005 -.108 .054 .048

Mother-Daughter .379 .181 .037 .318 .212 .135

Mother-Son .128 .188 .494 -.139 .104 .182

Father-Daughter .138 .230 .550 -.092 .172 .592

Father-Son -.137 .230 .550 -.457 .205 .026

*Table 4*. Two- and Three-Way Interactions Post-Hoc Analyses for Math Behavioral Intentions and Math Attitudes

Math Behavioral Intentions Math Attitudes

b S.E. *p* b S.E. *p*

2-Way interaction -.088 .038 .021 -.033 .043 .435*

( Dyad type X Child Math Anxiety)

Mother-Daughter .251 .130 .054

Mother-Son .061 .132 .643

Father-Daughter .129 .171 .450

Father-Son -.068 .137 .620

3-Way interaction -.040 .038 .287* -.135 .043 .002

Mother-Daughter .453 .155 .004

Mother-Son -.231 .083 .005

Father-Daughter .288 .197 .143

Father-Son -.453 .156 .004

*Note*. *Post-hoc analyses for non-significant interactions were not computed.

*Table 5*. Three-Way Interaction Post-Hoc Analyses for Math Devaluing

Math Devaluing

b S.E. *p*

3-Way interaction .050 .040 .074

Mother-Daughter -.246 .145 .090

Mother-Son -.120 .150 .424

Father-Daughter -.382 .184 .038

Father-Son -.136 .132 .303
